# Remarkable Quantity of Water Discharged by Paracentisis

**Published:** 1855-09

**Authors:** 


### REMARKABLE QUANTITY OF WATER DISCHARGED BY PARACENTISIS.

---

THE following has been communicated to us by Dr. GEORGE N. HOLMES, of Owensboro, Ky.:—"Below you will see a number of operations, for abdominal dropsy, upon a Mrs. Miles, the last five by the writer. The lady is still alive, but has removed to Missouri:

*March* 29, 1851, 88 pounds. *July* 25, 61 pounds. *Dec.* 9, 62 pounds.

*May* 1, 1852, 49 pounds. *Sept.* 11, 72 pounds.

*May* 17, 1853, 56 pounds. *August* 4, 47 pounds.

*Feb.* 3, 1854, 56 pounds.

The patient refused to be treated except by having paracentisis performed.

